# Supplementary material for: Actin-Depolymerizing Factor Gene Family Analysis Revealed That CsADF4 Increased the Sensitivity of Sweet Orange to Bacterial Pathogens
Source: Plants (Basel). 2023 Aug 25;12(17):3054. doi: 10.3390/plants12173054 (PMC10490069; doi:10.3390/plants12173054)
Supplement: Supplementary file 1 [file plants-12-03054-s001.zip › plants-2531988-supplementary.pdf]

Table S1. Ka/Ks replacement rate of tandem repeats of ADF gene in citrus.

Gene ID		Gene Name		Ka	Ks	Ka/Ks	EffectiveLen	AverageS-sites	AverageN-sites	cN	cS	pN	pS
Cs_ont_5g002950.1	Cs_ont_5g047810.1	CsADF1	CsADF3	0.14	2.70	0.05	417.00	87.83	329.17	41.92	64.08	0.13	0.73
Cs_ont_5g011920.1	Cs_ont_9g008180.1	CsADF2	CsADF7	0.07	1.12	0.06	429.00	96.00	333.00	22.08	55.92	0.07	0.58
Cs_ont_7g004070.1	Cs_ont_8g003160.1	CsADF4	CsADF5	0.08	1.38	0.06	417.00	89.08	327.92	24.83	56.17	0.08	0.63

Table S2. Primer sequence.

Gene	ID	Forward primer	Reverse primer
pCAMBIA1300s-CsADF4	Cs_ont_7g004070.1	GCTCACCATGGATCC ATGGCAAATGCAG-CATCTGGAA	CGCGAGCTCGGTACC ATTCGAAC-GGCTTCTCATGACATC
pCAMBIA-1132-CsADF4	Cs_ont_7g004070.1	GGCGGCCGCTCTAGAACTAGT ATGG-CAAATGCAGCATCTGGAA	GTCGACGGTATCGATAAGCTT ATTCGAAC-GGCTTCTCATGACATC
qCsADF1	Cs_ont_5g002950.1	TGACGAGTGCAAGCTCAAGT	TCCTCGTAGCTTGCTTGTGG
qCsADF2	Cs_ont_5g011920.1	AGCTACGAGGATTTCGCTGC	GCTCCCGTCGAAATCTGTCT
qCsADF3	Cs_ont_5g047810.1	CAACAGGTGACCGTCGAGAA	AATCATAGACGGCGTAGCGG
qCsADF4a	Cs_ont_7g004070.1	ATGCAGCATCTGGAATGGCA	ACAGCGTATCGGCACTCATC
qCsADF5	Cs_ont_8g003160.1	GAGGAGTGCCGATATGCTGT	CCTGTCCTTGGAGCTTGCATA
qCsADF6a	Cs_ont_8g027730.1	CCGGACGATGATTGCCGATA	GCTCTGATTCTCGACGCTGT
qCsADF7a	Cs_ont_9g008180.1	TTCACTGCATCTTTGCCGGA	GATTCGGGACACGTCAGGAG

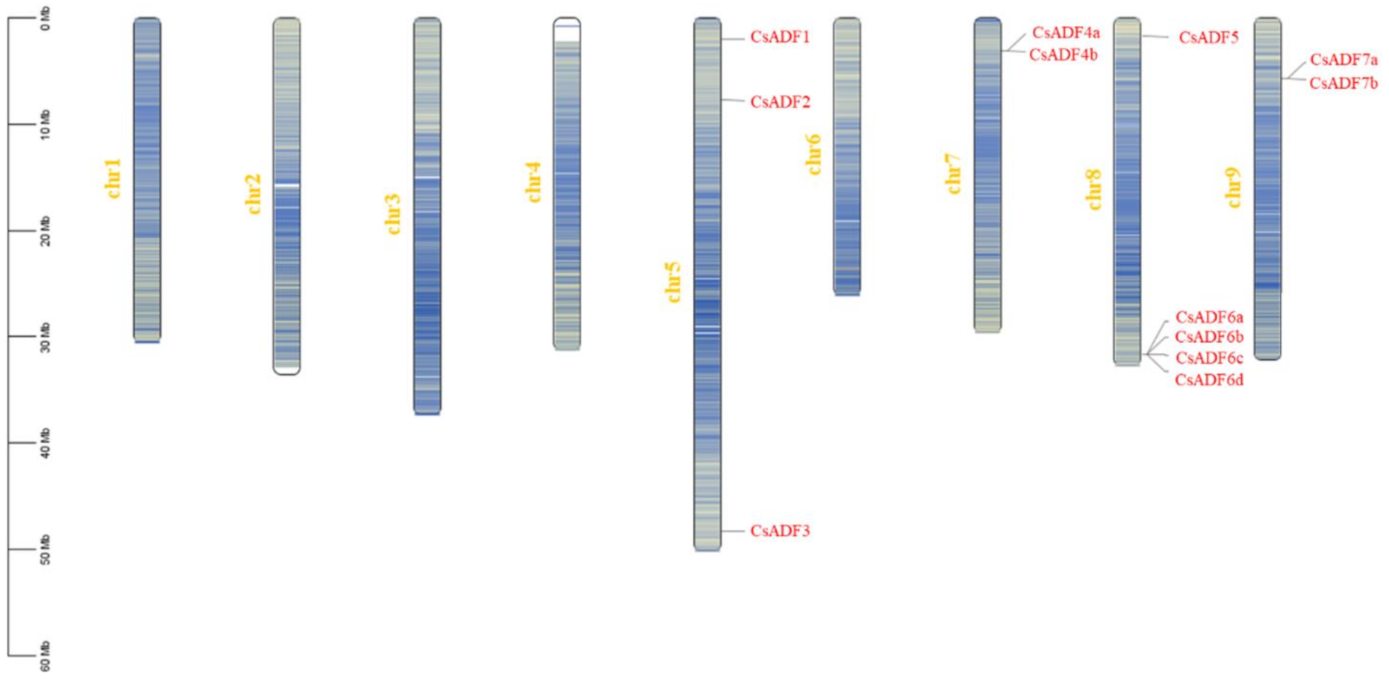

Figure S1. Chromosomal distributions of *CsADF* genes. Chromosomal names were placed at the left. The scale on left is in megabases (Mb). The red color represented high gene density , and the blue color represented low gene density .

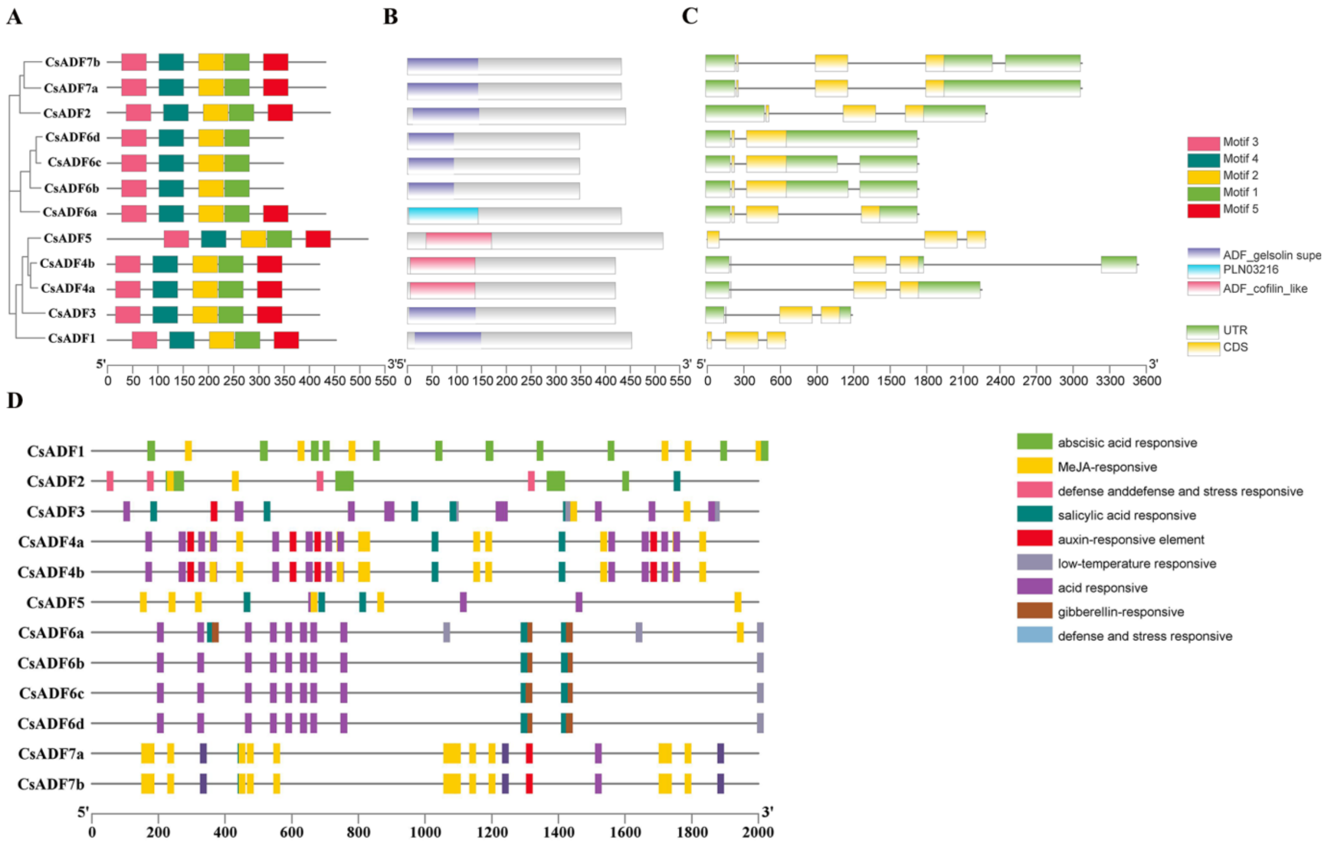

Figure S2. Phylogenetic relationship, exon-intron structures and motif composition of CsADFs. (A) Phylogenetic relationship of CsADFs and distribution of conserved motifs of CsADFs predicted by MEME tool. (B) (C) Exon-intron structures of CsADF genes. Exons-intron are indicated by wide color bar and black line, respectively. (D) Cis regulatory elements (CREs).
